# Viral Etiologies of Acute Respiratory Infections among Hospitalized Vietnamese Children in Ho Chi Minh City, 2004–2008

**DOI:** 10.1371/journal.pone.0018176

**Published:** 2011-03-24

**Authors:** Anh Ha Lien Do, H. Rogier van Doorn, My Ngoc Nghiem, Juliet E. Bryant, Thanh Hang thi Hoang, Quang Ha Do, Tan Le Van, Tan Thanh Tran, Bridget Wills, Vinh Chau van Nguyen, Minh Hien Vo, Cong Khanh Vo, Minh Dung Nguyen, Jeremy Farrar, Tinh Hien Tran, Menno D. de Jong

**Affiliations:** 1 Oxford University Clinical Research Unit, Wellcome Trust Major Overseas Program, Ho Chi Minh City, Vietnam; 2 Nuffield Department of Clinical Medicine, University of Oxford, Oxford, United Kingdom; 3 Department of Medical Microbiology, Academic Medical Center, University of Amsterdam, Amsterdam, The Netherlands; 4 Hospital for Tropical Diseases, Ho Chi Minh City, Vietnam; University of Hong Kong, Hong Kong

## Abstract

**Background:**

The dominant viral etiologies responsible for acute respiratory infections (ARIs) are poorly understood, particularly among hospitalized children in resource-limited tropical countries where morbidity and mortality caused by ARIs are highest. Improved etiological insight is needed to improve clinical management and prevention.

**Objectives:**

We conducted a three-year prospective descriptive study of severe respiratory illness among children from 2 months to 13 years of age within the largest referral hospital for infectious diseases in southern Vietnam.

**Methods:**

Molecular detection for 15 viral species and subtypes was performed on three types of respiratory specimens (nose, throat swabs and nasopharyngeal aspirates) using a multiplex RT-PCR kit (Seeplex™ RV detection, Seegene) and additional monoplex real-time RT-PCRs.

**Results:**

A total of 309 children were enrolled from November 2004 to January 2008. Viruses were identified in 72% (222/309) of cases, including respiratory syncytial virus (24%), influenza virus A and B (17%), human bocavirus (16%), enterovirus (9%), human coronavirus (8%), human metapneumovirus (7%), parainfluenza virus 1–3 (6%), adenovirus (5%), and human rhinovirus A (4%). Co-infections with multiple viruses were detected in 20% (62/309) of patients. When combined, diagnostic yields in nose and throat swabs were similar to nasopharyngeal aspirates.

**Conclusion:**

Similar to other parts in the world, RSV and influenza were the predominant viral pathogens detected in Vietnamese hospitalized children. Combined nasal and throat swabs are the specimens of choice for sensitive molecular detection of a broad panel of viral agents. Further research is required to better understand the clinical significance of single versus multiple viral coinfections and to address the role of bacterial (co-)infections involved in severe respiratory illness.

## Introduction

Acute respiratory illnesses (ARIs) are a leading cause of infectious disease-related morbidity, hospitalization, and mortality among children worldwide, particularly in developing countries and in young children (age < 5 years) [1]. Regardless of geographic location, the most common etiologic agents of ARIs in children are viruses [2]. The most frequently implicated viruses among hospitalized children are respiratory syncytial virus (RSV), human metapneumovirus (hMPV), influenza A and B viruses (InfV A and InfV B), parainfluenza viruses (PIV1-2-3) and adenoviruses (AdV) [3]. Other commonly implicated causes are human rhinoviruses (hRV), human coronaviruses (hCoV), enteroviruses (EnV) and human bocavirus (HBoV) [4], [5], [6], [7], [8].

The development of molecular methods such as conventional or real-time reverse transcriptase polymerase (RT-PCR) has facilitated rapid and sensitive simultaneous diagnostic detection of the variety of viruses causing respiratory tract infection [9]. However, limited resources and laboratory capacity precludes the routine use of molecular diagnostics in tropical lower-income countries such as Vietnam. As a consequence, insight into the aetiology of ARIs is lowest in regions of the world where morbidity and mortality are highest. Better understanding of the full spectrum of respiratory viruses causing ARIs in hospitalized patients in these settings is essential for improving preventive and therapeutic strategies and prioritizing diagnostic efforts.

Here we performed a 3-year prospective descriptive study to report on viral aetiologies, clinical features and epidemiological patterns of ARIs in hospitalized Vietnamese children using established commercially available and in-house nucleic acid amplification techniques. In addition, we used these techniques to evaluate which clinical specimen had the highest yield for ARI diagnostics by comparing nasopharyngeal aspirates and individual or combined throat and nasal swabs.

## Methods

### Study design

Patients were enrolled from November 2004 to January 2008 from the Paediatric Intensive Care Unit (PICU) and the Paediatric Respiratory Ward (PRW) at the Hospital for Tropical Diseases (HTD) in Ho Chi Minh City, the largest referral hospital for infectious diseases in southern Vietnam. Children were eligible for inclusion if less than 15 years of age and if admitted for an ARI or ARI-related condition (respiratory distress, pneumonia, bronchiolitis, croup) with an onset of illness less than 5 days before hospitalization. Excluded were patients who were discharged from a hospital in the previous 4 days, newborns who never left the hospital, patients with uncomplicated upper respiratory illness (e.g. rhinitis, sinusitis, otitis media), or patients with proven or suspected non-infectious respiratory symptoms (e.g asthma). Demographic, socio-economic, and clinical data were documented in case report forms (CRFs). Three types of respiratory specimens (nasal swabs, throat swabs, and nasopharyngeal aspirates) were collected on admission by trained personnel using standard operating procedures, and were placed in viral transport medium [10]. Specimens were kept at 4°C for a maximum of 24 h and then aliquotted and stored at −80°C until further processing.

### Ethics

The study was approved by the Scientific and Ethical Committee of the Hospital for Tropical Diseases and the Oxford University Tropical Research Ethical Committee.

Written informed consent was obtained from parents or legal guardians of children enrolled in the study.

### Diagnostic testing

For specimens collected between November 2004 to June 2007, RNA was extracted from 100 µl of each respiratory specimen using an inhouse GuSCN based extraction protocol [11] or Qiagen Viral RNA mini kits, according to the instructions of the manufacturer (Qiagen, Hilden, Germany). Since July 2007, an automated commercial GuSCN-based method was used, according the instructions of the manufacturer (Easy MAG 2.0, bioMérieux, Marcy l'Étoile, France). For each individual patient, all three respiratory specimens were extracted simultaneously. An internal RNA-virus control (equine arteritis virus (EAV)) was added to each sample prior to extraction at a standard concentration yielding a Ct value of 30 to 35 cycles in real-time RT-PCR. RNA was reversely transcribed using Superscript III reverse transcriptase (Invitrogen, Carlsbad, USA) and random hexamers (Roche, Mannheim, Germany). Each 20 µl reaction mixture contained 5 µl extracted RNA, 4 µl l of 5X RT-buffer, 0.5 mM of each dNTP (Roche), 2 ng random hexamer, 10 mM DTT (Invitrogen), 1 UI of RNAse inhibitor and 2 UI RT Superscript III. The cDNA synthesis was performed using an Eppendorf Master thermocycler gradient system (Perkin-Elmer Corporation, Foster City, USA) under the following conditions: 10 min at 25°C, 60 min at 50°C and 15 min at 75°C.

Detection of 12 respiratory viral pathogens, including influenza A (InfV A), influenza B (InfV B), RSV types A and B (RSV A, RSV B), hCoV (229E, OC43), hMPV, PIV types 1, 2 and 3 (PIV1, 2, 3), hRV A and AdV, was performed using a commercially available, internally controlled multiplex RT-PCR (Seeplex™ RV Detection kit, Seegene, Inc., Seoul, Korea) according to the manufacturer's instructions[12]. In addition, three previously described monoplex real-time PCRs were performed for detection of HBoV [7], enteroviruses (EnV) [13] and hCoV NL63[14] (**Table 1**).

**Table 1:** Primers and probes used in monoplex real-time RT-PCRs to detect HBoV, enterovirus and hCoV-NL63.

Virus name	Gene target	Primers/probes name	Nucleotide sequence
hBoV[7]	NP-1	Boca-F	5′-GGAAGAGACACTGGCAGACAA-3′
		Boca-R	5′-GGGTGTTCCTGATGATATGAGC-3′
		Boca probe	FAM-5′-CTGCGGCTCCTGCTCCTGTGAT-3′-TAMRA
EnV[13]	5′_NC	Entero-F	5′-CCCTGAATGCGGCTAAT-3′
		Entero-R	5′- ATTGTCACCATAAGCAGCC-3′
		Entero-probe	FAM-5′-CGGAACCGACTACTTTGGGT-3′-TAMRA
hCoV NL63[14]	Spike gene	NL63-F	5′ -GCGTGTTCCTACCAGAGAGGA-3′
		NL63-R	5′-GCTGTGGAAAACCTTTGGCA- 3′
		NL63-probe	FAM-5′-ATGTTATTCAGTGCTTTGGTCCTCGTGAT-3′-TAMRA

### Data analysis

A positive case was defined as the presence of any viral pathogen detected in any specimen type of a given patient. A patient was considered to have a single viral infection if only one pathogen was detected in one or more of the tested specimens. In case more than one viral pathogen detected, patients were considered to have viral co-infections. Severe disease was defined as being admitted to PICU. Fast breathing was classified according to WHO standards [15], i.e, ≥ 60 per minute in a child aged < 2 months, ≥ 50 per minute in a child aged 2 to 11 months and ≥ 40 per minute in a child aged ≥12 months.

We used a consensus standard to assess sensitivity: a patient was considered a true-positive (TP) for a given virus if any specimen type or testing method yielded a positive result. Thus, the specificity of each specimen type for any given virus was by definition 100%. Sensitivity per sample and virus was assessed using the following definitions: true negatives (TN), defined as the number of patients negative in all three specimens and false negatives (FN), defined as a negative sample while an alternative specimen from the same patient tested positive for a given virus. For the comparison of independent groups, we used the Mann-Whitney U test for continuous data and the Fisher exact test for categorical data. The Wilcoxon signed-rank test was used for pairwise comparisons of matched pairs of Ct values of the three types specimens from monoplex real-time RT-PCRs for HBoV, enteroviruses and hCoV NL63. Sensitivities of the different specimen types were compared using McNemar's test. All statistical tests were conducted at the two-tailed 5% significance level. Analyses were performed with R 2.9.1 (R Foundation for Statistical Computing, Vienna, Austria) and Intercooled Stata 9.2 (College Station, TX, USA).

## Results

### Study population

A total of 309 patients were enrolled in the study between November 2004 and January 2008. Ages ranged from 2 months to 13 years, and 181 (59%) patients were less than 2 years old. 79 (26%) of 309 patients were admitted to the PICU. Demographic and clinical characteristics of the PICU and PRW cohorts are shown in **Table 2**. At admission, most patients had fever, cough, runny nose, and fast breathing (154/309; 51%). Twenty percent (61/309) had indrawings, 33% (101/309) had wheezing, 31% (95/309) had crepitations, and 2% (6/309) had stridor. Patients with clinical symptoms suggestive of severe pneumonia [16], i.e fast breathing with cyanosis, indrawings or stridor, were all admitted to PICU. Oxygen support was required in 49% (39/79) of PICU patients, only one case required mechanical ventilation. Compared to PRW admitted patients, a significant higher number of PICU patients were hospitalized for more than 7 days (**Table 2**). In addition, they were more likely to have been exposed to cooking smoke and more often had other family members who were sick at home. A history of previous hospitalizations for respiratory illness was observed more frequently in patients admitted to PRW (**Table 2**).

**Table 2:** Demographic and clinical characteristics of study cohort.

	PICU		PRW	
	N	Value(%)	N	Value(%)
Demographic				
Median age in months (IQR)	78	15.5 (10–25)	230	24 (17–36)
Infant (<12months)	78	30 (38.0)	230	26 (11)
Male	79	44 (56)	223	128 (57)
Mean birth weight (kg) (IQR)	74	3.2 (2.9–3.4)	171	3.1 (2.8–3.4)
Median number of family members (IQR)	78	5 (4–7)	222	5 (4–6)
**Exposed to cooking smoke** 1	79	**69 (87)**	230	**45 (20)**
Living with smokers	79	60 (76)	230	165 (81)
Medical story and clinical characteristics				
**Previous hospitalization with respiratory diseases 2**	76	**27 (36)**	226	**120 (53)**
**Other family members sick at home 3**	78	**20 (26)**	226	**30 (13)**
**Median (IQR) onset of illness 4**	79	**4 (3–5)**	229	**3 (3–4)**
**Fast breathing 5**	77	**55 (71)**	224	**102 (46)**
Cyanosis	79	4 (5.)	228	0 (0)
Indrawings	79	61 (77)	228	0 (0)
Stridor	78	5 (6)	227	1 (0)
**Wheezing 6**	79	**65 (82)**	226	**36 (16)**
**Crepitations 7**	79	**55 (70)**	227	**40 (18)**
Fever (>37.5°C)**Fever ≥ 38.5°C 8**	79	77 (97)**25 (32)**	229	229 (100)**159 (69)**
Rash	79	1 (1)	227	17 (7)
Cough	79	77 (97)	229	226 (99)
Runny noses	76	49 (61)	228	220 (96)
Oxygen	79	39 (49)	229	0 (0)
Diagnosis at admission				
Bronchiolitis	79	24 (30)	230	81 (35)
**Broncho-pneumonia 9**	79	**42 (53)**	230	**6 (3)**
Pneumonia	79	10 (13)	230	0 (0)
Laryngitis	79	3 (4)	230	1 (0)
ARIs	79	0 (0)	230	142 (62)
Median of duration of hospitalization (IQR)**Duration of hospitalization >7 days 10**	78	7 (6–9)**34 (44)**	218	6 (4–7)**44(20)**
Median of number of white cells in blood (K/mm^3^) (IQR)	79	14.2 (10.0–19.5)	223	11.1 (7.9–15.0)
Clinical outcomes				
Fully recovery (%)	78	69 (88)	217	205 (94)
Death	78	2 (3)	217	0 (0)
Viral pathogen				
Virus positive (%)	79	58 (73.4)	230	164 (71.3)
Single infection (%)	79	43 (54.4)	230	117 (50.9)
Co-infection (%)	79	15 (19.0)	230	47 (20.4)
Single RSV infection (%)	79	14 (17.7)	230	38 (16.5)
Single Influenza virus infection (%)	79	5 (6.3)	230	25 (10.9)
Single HBoV infection (%)	79	6 (7.6)	230	17 (7.4)
Single hMPV infection (%)	79	4 (5.1)	230	12 (5.2)
Single EnV infection (%)	79	2 (2.5)	230	11 (4.8)
Single hRV A infection (%)	79	4 (5.1)	230	1 (0.4)

PICU, Pediatric Intensive Care Unit; PRW, Pediatric Respiratory Ward. Values in bold and underline indicate statistical significance (p< 0.05).

1 p value Fisher's exact  =  0.001.

2 p value Fisher's exact  =  0.008.

3 p value Fisher's exact  =  0.01.

4 p value Mann-Whitney'test  = 0.003.

5, 6, 7 Fast breathing, wheezing and crepitations (p value Fisher's exact  =  0.001).

8 p value Fisher's exact  =  0.001.

9 p value Fisher's exact  =  0.001.

10 p value of Fisher's test  = 0.001.

Diagnoses at admission were based on clinical and laboratory information and Xray interpretation; physicians were unaware of diagnostic results for respiratory viruses during data collection, except for two suspected H5N1 cases as these were diagnosed for public health purposes by specific PCR. Nearly half of cases (46%, 142/309) were diagnosed as ARI at admission, followed by bronchiolitis in 34%, broncho-pneumonia in 16%, pneumonia in 3% and laryngitis or laryngotracheitis in 1% (4/309). Broncho-pneumonia was more frequently diagnosed among PICU than PRW patients (**Table 2**).

The median duration of hospitalisation was 6 days, and 25% of patients were hospitalized for more than 7 days. Discharge information was available for 295 of 309 cases: 93% of patients fully recovered; 1% (4/295) had an incomplete recovery at the time of discharge; 5% (15/295) went home without permission of ward doctors; and 2 patients (1%) died. One fatal case was diagnosed with influenza A (H5N1)-associated viral pneumonia and died on day of admission. The second fatal case was negative for all viruses tested, and was diagnosed with severe pneumonia with shock syndrome of unknown aetiology. Diagnosis of bacterial pathogens was not systematically performed. However, 98% of patients received antibiotic treatment during admission. The two suspect cases of Influenza A/H5N1 infection received oseltamivir treatment.

### Viral etiologies, age distribution, seasonality and severity

One or multiple viral pathogens were detected in respiratory specimens of 222 of 309 (72%) patients. RSV was most frequently detected (24%, 73/309), followed by influenza viruses (17%, 51/309), HBoV (16%, 50/309) and enteroviruses (9%, 28/309) (**Table 3**). Single infections accounted for 52% (160/309) of cases. Infections with multiple viruses were found in 20% (62/309): dual infections were identified in 18% of cases (55/309), triple infections in 2% (6/309), while in one case 4 different viruses were detected.

**Table 3:** Viral etiologies identified.

	Single infection			Co- infection			Total positive cases
Viral causes	PICU	PRW	Subtotaln (%)	PICU	PRW	Subtotaln (%)	n (%)
RSV	14	38	52 (17)	6	15	21 (7%)	73 (24)
RSV A	7	23	30 (10)	3	9	12 (4)	42 (14)
RSV B	7	15	22 (7)	3	6	9 (3)	31 (10)
Influenza	5	25	30 (10)	5	16	21 (7)	51 (17)
InfV A	2	15	17 (6)	2	8	10 (3)	27 (9)
InfV B	3	10	13 (4)	3	8	11 (4)	24 (8)
HBoV	6	17	23 (7)	6	21	27 (9)	50 (16)
EnV	2	11	13 (4)	3	12	15 (5)	28 (9)
hMPV	4	12	16 (5)	1	4	5 (2)	21 (7)
Parainfluenza virus	4	7	11 (3)	2	6	8 (3)	19 (7)
PIV-1	1	3	4 (1)	1	3	4 (1)	8 (3)
PIV-2	1	0	1 (0)	0	2	2 (1)	3 (1)
PIV-3	2	4	6 (2)	1	1	2 (1)	8 (3)
Coronavirus	4	3	7 (2)	5	12	17 (5)	24 (8)
hCoV-NL63	3	2	5 (2)	5	11	16 (5)	21 (7)
hCoV-229E	1	0	1 (0)	0	0	0 (0)	1 (0)
hCoV-OC43	0	1	1 (0)	0	1	1 (0)	2 (1)
AdV	0	3	3 (1)	3	9	12 (4)	15 (5)
hRV A	4	1	5 (2)	1	5	6 (2)	11 (4)
Total	43	117	160 (52)	32	100	62 (20)	222 (72)

Case numbers reflect combined results from all three specimen types. All percentages were calculated as the fraction of the total study population (N = 309).

RSV and influenza viruses were the leading causes of single infections, followed by hBoV and hMPV. Human bocavirus was most frequently involved in co-infections (n = 27), followed by RSV, influenza viruses (both n = 21), hCoV-NL63 (n = 16) and enteroviruses (n = 15). Among RSV cases, co-infection with hBoV was most frequent (8/21), followed by influenza viruses (7/21) and hCoV (5/21). For influenza cases, co-infection with hCoV was most frequent (8/21), followed by RSV (7/21) and HBoV (6/21). 16/21 additional viruses were also detected among hCoV-NL63 infection cases.

RSV was the most common virus detected in children less than 5 years whereas influenza virus was most prevalent virus among children over 5 (**Table 4**). Wheezing was significantly more frequent among RSV-positive cases (32/72 (44%)) than among RSV-negative cases (69/233 (30%); p = 0.02), as were crepitations (36/72 (50%) vs. 59/175 (25%); p = 0.001). Among HBoV-infected children, throat viral loads were higher in patients with wheezing symptoms than in those without, as indicated by lower Ct values in the former (p =  0.03).

**Table 4:** Age group distribution of viral etiologies.

	Age groups					
Virus detected	<1 year (N = 56)		1-5 years (N = 239)		>5 years (N = 13)	
	n	%	n	%	n	%
RSV A& B	12	21%	60	25%	1	8%
InfV A&B	6	11%	41	17%	4	31%
HBoV	12	21%	36	15%	1	8%
EnV	5	9%	23	10%	0	0%
hMPV	0	0%	20	8%	1	8%
PIV (1-3)	8	14%	11	5%	0	0%
hCoV	5	9%	16	7%	3	23%
AdV	1	2%	14	6%	0	0%
hRV A	2	4%	9	4%	0	0%
Single infections	28	50%	126	53%	5	38%
Co-infections	10	18%	50	21%	2	15%
Positives cases	38	68%	176	74%	7	54%

Percentages were calculated based on the fraction of study population within each age group. (Note, age data was not available for one case.)

Except for RSV, influenza and HBoV, no significant associations were observed between viral etiologies and age distributions, signs or symptoms, clinical diagnosis, or duration of hospital stay (**Table 2** and **Table 5**). Similarly, there were no apparent differences in clinical characteristics or severity between single and multiple infections, nor between virus-positive and virus-negative cases. However, among virus -positive patients, we found that PICU patients were significantly younger than respiratory ward patients (Mann-Whitney's test p =  0.001).

**Table 5:** Summary of associations between clinical characteristics and viral single/co-infection or viral positive cases and viral negative cases.

Characteristic	Single infection (n = 160)	Co-infection (n = 62)	Virus positive (n = 222)	Virus negative (n = 87)
**Age** a	21 (14–36)	24 (14–35)	22 (14–35)	24 (14–36)
**Fever (%)**	99 (61.9)	35 (56.5)	134 (60.4)	50 (57.5)
**Fast breathing** b **(%)**	84 (52.5)	28 (45.2)	112 (50.5)	45 (51.7)
**Cyanosis (%)**	3 (1.9)	0 (0)	3 (1.4)	1 (1.1)
**Oxygen (%)**	22 (13.8)	5 (8.1)	27 (12.2)	12 (13.8)
**Indrawing (%)**	34 (21.3)	9 (14.5)	43 (19.4)	18 (20.7)
**Stridor (%)**	2 (1.3)	2 (3.2)	4 (1.8)	2 (2.30
**Wheeze (%)**	58 (36.3)	18 (29.0)	76 (34.2)	25 (28.70
**Crepitations (%)**	58 (36.3)	13 (21.0)	71 (32.0)	24 (27.6)
**Bronchiolitis (%)**	59 (36.9)	16 (25.8)	75 (33.8)	30 (34.5)
**Broncho-pneumonia (%)**	32 (20.0)	9 (14.5)	41 (18.5)	7 (8.0)
**Pneumonia (%)**	3 (1.9)	2 (3.2)	5 (2.3)	5 (5.7)
**Laryngitis (%)**	1 (0.6)	2 (3.2)	3 (1.40	1 (1.0)
**ARIs (%)**	65 (40.6)	33 (53.2)	98 (44.1)	44 (50.6)
**Hospital duration** c	7(5–8)	6(4–8)	6(5–8)	6(5–8)
**Duration > 7 days** d **(%)**	40 (25.0)	15 (24.2)	55 (24.8)	23 (26.4)

Percentages were calculated based on the fraction of patients having a specific symptom within each group.

a Median (IQR) of age of each group.

b fast breathing was defined according to the standard WHO [15].

c Median (IQR) of duration of hospitalization (days).

d Number and percentage of patients having duration of hospitalization lasted longer than 7 days.

**Figure 1:**
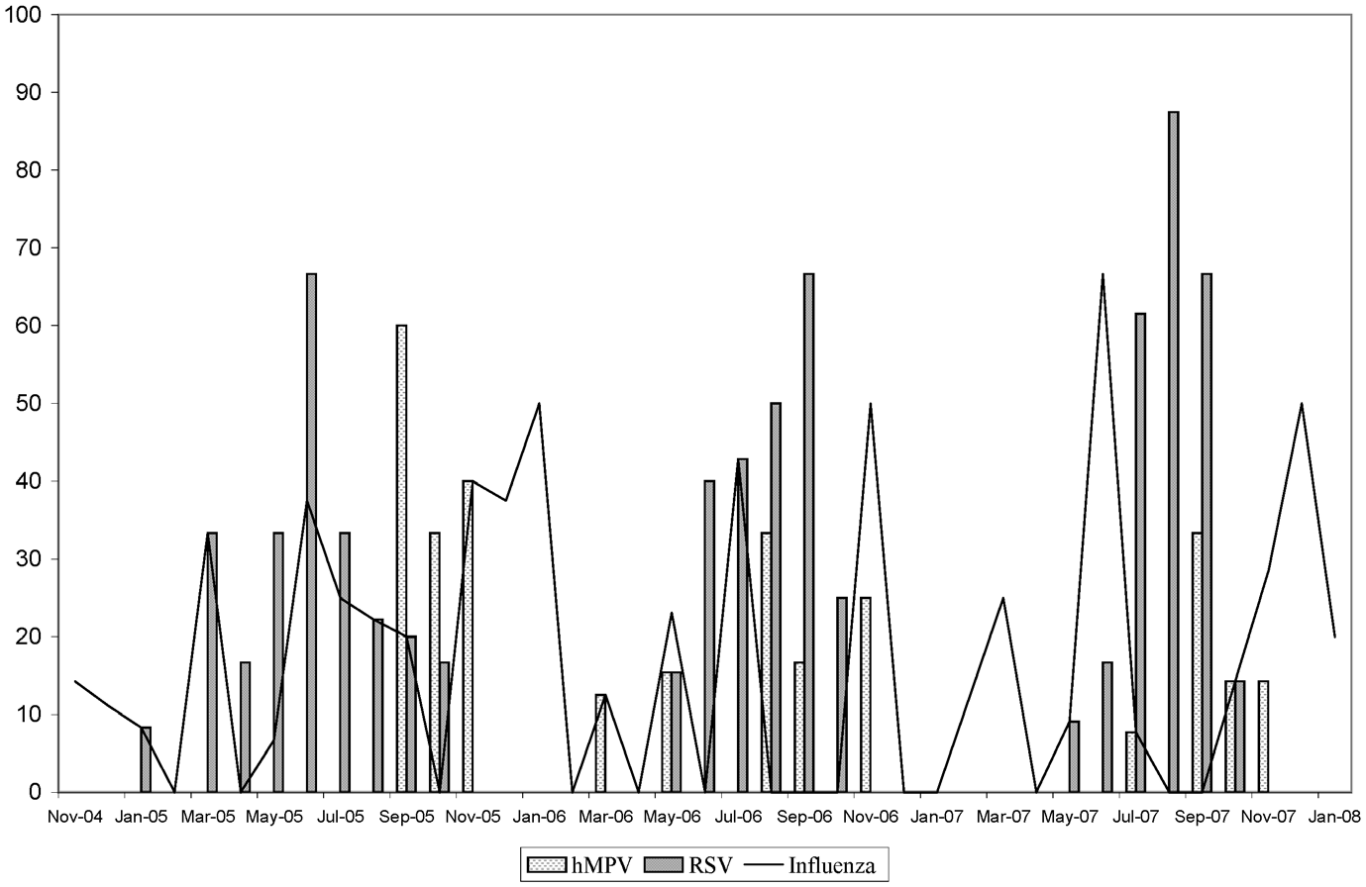
Proportion of RSV and hMPV positive cases recruited each month from November 2004 to January 2008. Time in months is displayed on the X-axis and the percentage of positive cases of each virus among all cases recruited in that month on the y-axis.

RSV infection showed seasonal variation with peaks during the rainy season from May to October. Seasonality of hMPV infection was also apparent during the rainy season. Influenza cases occurred throughout the year (**Figure 1**). The numbers of remaining viral aetiologies were insufficient to detect seasonal patterns.

### Optimal specimen for viral diagnosis

Virus was detected in a total of 226 respiratory specimens from 222 patients. 117 (40%) patients tested positive in all three specimens collected, 33 (11%) were positive only in nasal swabs, 29 (10%) only in throat swabs, and 46 (16%) only in NPA specimens. The diagnostic yield of the three specimen types are shown in **Table 6**. Overall, diagnostic yield from NPAs was superior although pair-wise differences were not significant. However, separate analyses of specific viruses demonstrated significantly superior yield of NPA specimens for influenza viruses (p = 0.05 and p = 0.006 for NPA versus nasal swab and throat swab, respectively). For enteroviruses, throat swabs were superior (p = 0.01 and p = 0.02 for throat swab versus nasal swab and NPA, respectively). In addition, Ct values of enteroviruses from throat swabs were significantly lower, indicating higher viral loads, compared to nasal swabs (median Ct value of throat swab was 30.8 (IQR 28.2–33.7) versus median Ct value of nasal swab 34.6 (IQR 33–37.1), p  =  0.003).

**Table 6:** Diagnostic sensitivity and efficacy of respiratory specimens and combined nasal-throat (NT) swabs by viral etiology.

Virus detected	Total positive cases (N)	Nasal swabs		Throat swabs		NPA		NT swabs	
		Sensitivity % (n)	95%CI	Sensitivity % (n)	95%CI	Sensitivity % (n)	95%CI	Sensitivity % (n)	95%CI
Any virus*	222	75(166)	69–81	73(161)	67–78	78(173)	73–83	79(176)	73–84
RSV	73	84(61)	75–92	81(59)	72–90	90(66)	84–97	93(68)	85-98
InfV	51	**53** i(27)	39–67	**45** ii(23)	31–59	**75** i,ii(38)	63–87	73(37)	58-84
HBoV	50	65(32)	51–78	52(26)	38–66	65(32)	52–79	78(39)	64-89
EnV	28	**50** iii(14)	32–69	**85**_iii,iv(23)	72–99	**48** iv, v(13)	29–67	**93** v(26)	77-99
hMPV	21	67(14)	47–87	71(15)	52–91	71(15)	52–91	81(17)	58-95
PIV	19	74(14)	49–91	44(8)	22–67	72(13)	52–93	79(15)	54-94
AdV	15	73(11)	51–96	87(13)	70 -100	73(11)	51–96	100(15)	78-1
hRV A	11	64(7)	35–92	73(8)	46–99	91(10)	74–100	82(9)	48-98
hCoV	24	58(14)	39–78	58(14)	39–78	50(12)	30 -70	83(20)	63-95
Picornavirus#	36	58(21)	41–75	83(30)	67–91	64(23)	46–79	92(33)	78-98

Underlined and bold numbers indicate significant differences in paired values by McNemar's test:

i between nasal swabs and NPA (p = 0.05);

ii between NPA and throat swabs (p = 0.006);

iii between nasal swabs and throat swabs (p = 0.01);

iv between throat swabs and NPA (p = 0.02);

v between NT swabs and NPA ( = 0.001).

*refers to the total number of positive cases, defined as the detection of any viral pathogen in any specimen per patient.

# refers to cases in whose samples enterovirus or human rhinovirus A was detected.

We evaluated our dataset to examine the diagnostic yield from a combination of the results of nasal and throat swabs (NTS) relative to NPA (**Table 6** **)**. Our analysis indicated that testing of combined NTS should yield comparable sensitivities to NPAs for detection of all respiratory viruses of our panel, and is likely to significantly improve the detection of enteroviruses (McNemar's test p  =  0.001).

## Discussion

Here we report the viral etiologies of ARIs in 309 hospitalized children in southern Vietnam enrolled during a period of more than three years (11/2004-1/2008). Seventy-two percent of patients were diagnosed with single virus infections and 20% were co-infected with multiple respiratory viruses. Overall, RSV was the most frequently detected virus, and accounted for 24% of infections in children less than 5 years old. Influenza (17%) was the second most common virus detected, while the recently discovered hBoV, hMPV and hCoV-NL63 were detected in 16%, 7%, and 7% of cases, respectively. Enteroviruses were found in 9% of cases, supporting studies from both Europe [6], [17] and southeast Asia [18] which also detected enteroviruses in a large fraction of children with respiratory infections.

Our findings are consistent with other reports from Asia and elsewhere indicating that RSV and influenza are dominant causes of severe respiratory tract infections in children [19], [20], [21], [22]. However, the clinical significance of co-infections and the relative ranking of the other respiratory viruses in our panel remain unclear. A recent study of ARI in Nha Trang (Central Vietnam) reported almost identical prevalence rates for RSV and influenza A but found a lower percentage of co-infections (11% versus 20% in our study) and different prevalence rates for rhinovirus (28% versus 4% in our patients) and bocavirus (5% verus 16% in our patients) [23], [24]. These differences likely reflect differences in study design and testing protocols: Yoshida *et al.* included a large number of ambulatory outpatients whereas our study focused exclusively on hospitalized cases; their diagnostic testing was based on nasal swab alone; their PCR for rhinovirus was able to detect rhinovirus types A, B and C whereas ours detected only rhinovirus A and B; and their panel of viruses did *not* include either enteroviruses or hCoV NL63.

We found that RSV and hMPV cases were detected mainly during the rainy season from May to October, supporting previous observations from tropical or subtropical regions [23], [24]. In contrast, influenza seems to occur throughout the year with no discernable peak incidences.

Our results confirm and extend previous observations regarding the importance of RSV in children under 5 [23], [24], and the clinical association between wheezing and RSV infection [25] [26], [27]. Increasing evidence suggests that RSV infections may be related to asthma phenotypes, with progressive disappearance of this effect with increasing age [28]. As such, our findings emphasize the importance of screening for RSV in paediatric cases of asthma. Our results also confirm observations of Allander *et al* suggesting an association between HBoV levels and symptoms of wheezing [7], indicating that information about viral load may be important for better understanding of disease pathogenesis.

We found significant associations between disease severity and a history of exposure to household cooking smoke. Indoor pollution due to biomass fuels (wood, crop residues and animal dung) or coal burning is a known risk factor for ARI mortality and morbidity in developing countries[29], [30]. Disease severity was also associated with longer delays between onset of illness and presentation to hospital. Delays in presentation are typical of health seeking behavior in lower income households of developing countries [31]. Our patient survey questionnaire did not include explicit questions regarding household income, however our findings clearly indicate the need for further research on the socio-economic risk factors associated with ARI.

Numerous previous studies have investigated optimal sampling methods for diagnosis of respiratory viruses by culture, immunofluorescence, or molecular techniques [32], [33], [34], [35], [36], [37]. Regardless of the diagnostic approach, NPA specimens typically exhibit increased sensitivity (15-31%) relative to nasal swabs or throat swabs. However, the differential increase appears less marked for molecular based methods [32]. Indeed, results of the present study indicate that, although NPAs yielded the highest overall yield of virus detection (78%, 173/222), the differential improvement over nasal or throat swabs was marginal and not statistically significant across all viruses (**Table 6**). Nevertheless, NPAs were significantly superior to nasal or throat swabs for detection of influenza viruses (p≤0.05), whereas throat swabs were superior for enteroviruses (p≤0.05). Combining the results of nasal and throat swabs rendered comparable sensitivities to NPAs for detection of all respiratory viruses of our panel and increased sensitivity for enteroviruses. As nasal and throat swabs are easier to obtain and less distressing for patients, these samples are preferred in our setting.

**Figure 2:**
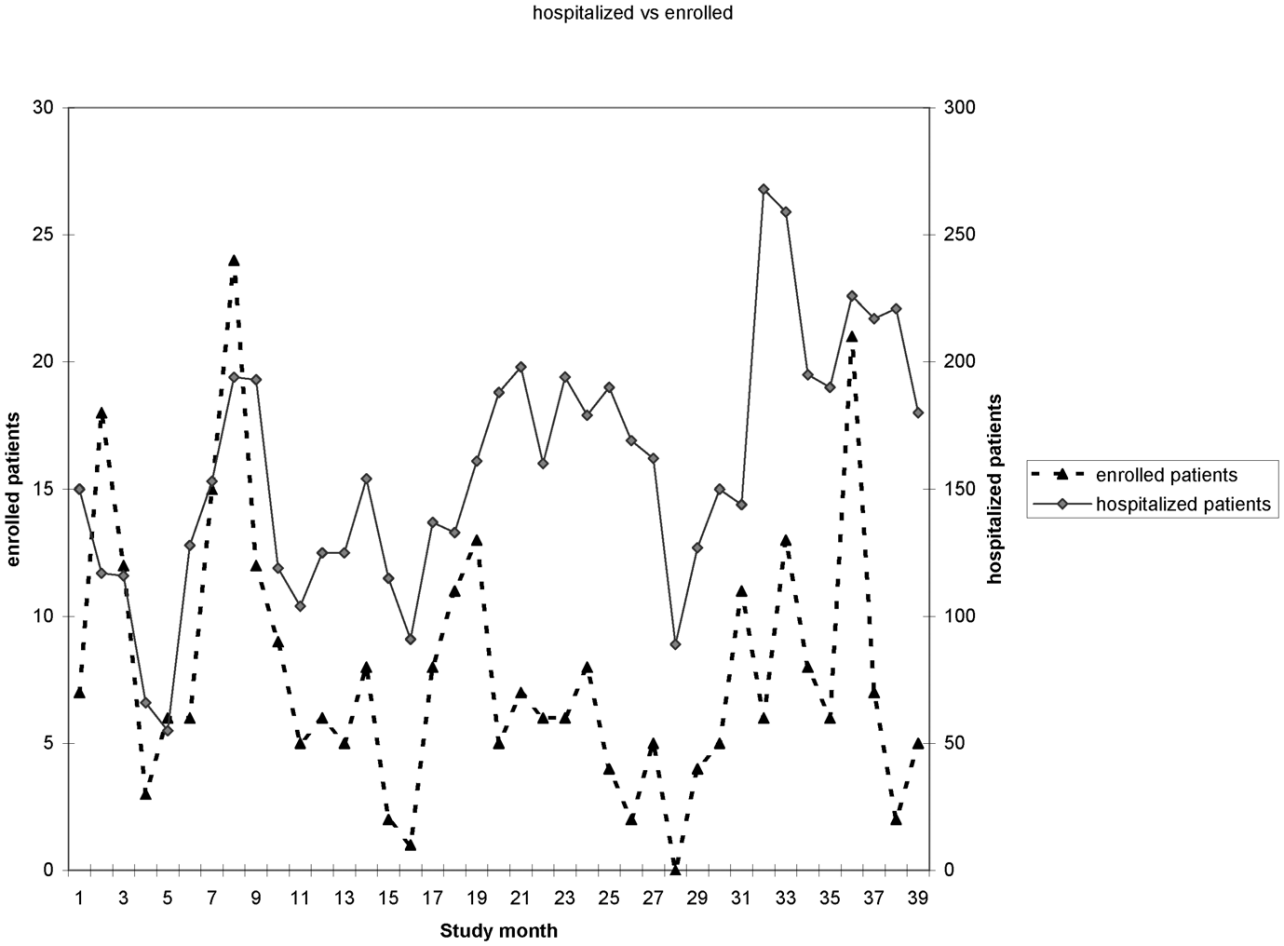
Number of cases enrolled and total numbers of ARI children hospitalized in HTD, November 2004 to January 2008.

There were several limitations to our study. Firstly, we focused only on viral aetiologies since these are common causes of ARI and understudied in this region. However, this prevented the possibility of addressing key questions about bacterial pathogens and the possible role of viral and bacterial co-infections. As nearly all children in our study received antibiotic treatment, and issues involving judicious use of antibiotics and resistance development is becoming increasingly important in Vietnam, inclusion of bacterial pathogens in future studies will be essential. Second, our testing algorithm did not include measles virus. We suspect there may have been undiagnosed measles cases within our cohort, since 6/18 cases who presented with rash on admission were negative for all viruses tested; furthermore, national surveillance data indicates that Vietnam experienced a rise in measles cases in 2005–2006 [38]. Lastly, our sample size was limited and insufficient to allow more refined observations regarding differences in age distributions, clinical characteristics, or determinants of severity between specific viral species. The principle reasons for limited enrollment were a) resource constraints and feasibility (overburdening of doctors and nurses in wards where admission of 2 to 3 patients per bed is common practice), and b) limited familiarity with clinical research among patients and parents in this setting and thus additional challenges to obtaining consent. In total, during the 3-year study period, only 4.1% of all admitted ARI patients at the respiratory ward (40 beds) were enrolled in the study, and 14.2% of ARI patients admitted to the PICU (15 beds). While the sample size of enrolled patients relative to the total number of admitted patients was small, the proportion remained stable throughout the study period and the number of enrolled patients followed a similar pattern as the number of admitted patients over time (**Figure 2**). For these reason, we believe that our study population nevertheless provided a reasonable representation of the overall ARI patient population in Ho Chi Minh City at the time.

In conclusion, our study contributes critical baseline epidemiological data on ARI in Vietnam, and highlights the importance of RSV and influenza as dominant viral etiologies of severe pediatric ARI. Our findings indicate that combined nasal-throat swabs are the specimens of choice for sensitive molecular detection of a broad panel of viral agents. Pneumonia remains a leading cause of death among children less than 5 years old in developing countries, and continues to be a salient public health problem in Vietnam. Enhancing existing surveillance systems to better understand disease burden of respiratory pathogens is one step forward to development of therapeutic and prevention strategies.
